# Supplementary material for: Serum Fatty Acids and Inflammatory Patterns in Severe Obesity: A Preliminary Investigation in Women
Source: Biomedicines. 2024 Oct 3;12(10):2248. doi: 10.3390/biomedicines12102248 (PMC11505423; doi:10.3390/biomedicines12102248)
Supplement: Supplementary file 1 [file biomedicines-12-02248-s001.zip › Supplementary Table S3.pdf]

Supplementary Table S3. Correlations between omega 3 and 6 fatty acids and CRP

Variables	r	p-value
Omega 6		
C18:2n6	-0.21	0.17
C18:3n6	0.38	**0.01**
C20:2n6	0.04	0.76
C20:3n6	-0.19	0.21
C20:4n6	0.26	0.09
C22:2n6	-0.03	0.81
Omega 3		
C18:3n3	0.38	**0.01**
C18:4n3	0.10	0.52
C20:3n3	0.06	0.69
C20:4n3	0.40	**0.00**
C20:5n3	0.10	0.51
C22:6n3	0.05	0.75

$p < 0.05$ was considered statistically significant.
