# Supplementary material for: Serum Fatty Acids and Inflammatory Patterns in Severe Obesity: A Preliminary Investigation in Women
Source: Biomedicines. 2024 Oct 3;12(10):2248. doi: 10.3390/biomedicines12102248 (PMC11505423; doi:10.3390/biomedicines12102248)
Supplement: Supplementary file 1 [file biomedicines-12-02248-s001.zip › Supplementary Table S2.pdf]

Supplementary Table S2. Correlations between fatty acid profile and Adipo/Lep ratios in women with severe obesity.

Variables	r	p-value
Saturated (SFA) %by area		
C14:0	0.03	0.81
C16:0	-0.09	0.56
C20:0	0.15	0.92
C22:0	-0.11	0.49
C14:1C	0.03	0.81
C16:1n7	-0.03	0.82
C18:1n9	-0.05	0.75
C18:1n7	-0.03	0.82
C20:1n9	-0.07	0.65
Omega 6		
C18:2n6	0.01	0.93
C18:3n6	0.15	0.34
C20:2n6	0.00	0.98
C20:3n6	-0.12	0.42
C20:4n6	-0.01	0.94
C22:2n6	-0.00	0.97
Omega 3		
C18:3n3	0.08	0.62
C18:4n3	0.17	0.28
C20:3n3	-0.10	0.53
C20:4n3	0.02	0.87
C20:5n3	0.19	0.23
C22:6n3	0.74	**0.00**

$p < 0.05$ was considered statistically significant.
