# Supplementary material for: Serum Fatty Acids and Inflammatory Patterns in Severe Obesity: A Preliminary Investigation in Women
Source: Biomedicines. 2024 Oct 3;12(10):2248. doi: 10.3390/biomedicines12102248 (PMC11505423; doi:10.3390/biomedicines12102248)
Supplement: Supplementary file 1 [file biomedicines-12-02248-s001.zip › Supplementary Table S1.pdf]

Supplementary Table S1. Correlations between fatty acid profile and Lep/Adipo ratios in women with severe obesity

Variables	r	p-value
Saturated (SFA) %by area		
C14:0	-0.00	0.97
C16:0	0.92	0.56
C20:0	-0.01	0.93
C22:0	-0.17	0.27
Monounsaturated(MUFA) %by area		
C14:1C	-0.02	0.89
C16:1n7	-0.06	0.66
C18:1n9	0.07	0.62
C18:1n7	0.05	0.75
C20:1n9	-0.09	0.53
Polyunsaturated (PUFA) %by area		
Omega 6		
C18:2n6	0.00	0.96
C18:3n6	0.04	0.76
C20:2n6	0.00	0.97
C20:3n6	0.52	0.00
C20:4n6	0.03	0.83
C22:2n6	0.03	0.83
Omega 3		
C18:3n3	-0.16	0.31
C18:4n3	-0.11	0.47
C20:3n3	-0.04	0.80
C20:4n3	0.06	0.68
C20:5n3	-0.13	0.39
C22:6n3	-0.23	0.14

$p < 0.05$ was considered statistically significant.
